# Homology-Based Interactions between Small RNAs and Their Targets Control Dominance Hierarchy of Male Determinant Alleles of Self-Incompatibility in *Arabidopsis lyrata*

**DOI:** 10.3390/ijms22136990

**Published:** 2021-06-29

**Authors:** Shinsuke Yasuda, Risa Kobayashi, Toshiro Ito, Yuko Wada, Seiji Takayama

**Affiliations:** 1 Division of Biological Sciences, Nara Institute of Science and Technology, Nara 630-0192, Japan; shinsuke-yasuda@takii.co.jp (S.Y.); kobayashi.risa.ki7@bs.naist.jp (R.K.); itot@bs.naist.jp (T.I.); 2 Department of Applied Biological Chemistry, Graduate School of Agricultural and Life Sciences, The University of Tokyo, Tokyo 113-8657, Japan

**Keywords:** dominance hierarchy, self-incompatibility, epigenetics, small RNA, Brassicaceae, *Arabidopsis lyrata*

## Abstract

Self-incompatibility (SI) is conserved among members of the Brassicaceae plant family. This trait is controlled epigenetically by the dominance hierarchy of the male determinant alleles. We previously demonstrated that a single small RNA (sRNA) gene is sufficient to control the linear dominance hierarchy in *Brassica rapa* and proposed a model in which a homology-based interaction between sRNAs and target sites controls the complicated dominance hierarchy of male SI determinants. In *Arabidopsis halleri*, male dominance hierarchy is reported to have arisen from multiple networks of sRNA target gains and losses. Despite these findings, it remains unknown whether the molecular mechanism underlying the dominance hierarchy is conserved among Brassicaceae. Here, we identified sRNAs and their target sites that can explain the linear dominance hierarchy of *Arabidopsis lyrata*, a species closely related to *A. halleri*. We tested the model that we established in *Brassica* to explain the linear dominance hierarchy in *A. lyrata*. Our results suggest that the dominance hierarchy of *A. lyrata* is also controlled by a homology-based interaction between sRNAs and their targets.

## 1. Introduction

Almost half of all angiosperms have a self-incompatibility (SI) system to avoid self-fertilization which helps maintain genetic diversity within the species. Most Brassicaceae plants, including *Arabidopsis lyrata*, have SI systems ensuring that the plants’ sporophytic stigmas reject their own pollen grains. This SI reaction is genetically controlled by a single multiallelic locus called the *S* locus, which contains the pollen and stigma determinant genes *S-LOCUS PROTEIN 11* (*SP11*, also called *SCR*) [1,2,3] and *S-LOCUS RECEPTOR KINASE* (*SRK*) [4,5], respectively. The SI reaction in *Brassica rapa* is caused by the *S* haplotype-specific direct interaction between SP11 and SRK in the stigma epidermis [6,7]. These two genes are conserved in members of the Brassicaceae family, such as *Brassica oleracea* [4], *A. lyrata* [8], and *Arabidopsis halleri* [9].

*B. rapa* has a complicated dominance hierarchy among *SP11* alleles, which are expressed sporophytically in anther tapetum cells. Based on analysis of pollen phenotypes, class I (*S_8_*, *S_9_*, *S_52_*, *S_12_*) *SP11* alleles are dominant over class-II alleles (*S_44_*, *S_60_*, *S_40_*, *S_29_*) [10]. In addition, class-II alleles follow a linear dominance hierarchy: *S_44_* > *S_60_* > *S_40_* > *S_29_* [10]. This complex hierarchy is controlled by interactions between just two sRNAs, *SP11 methylation inducer* (*Smi*) and *Smi2*, and their targets [11,12]. These sRNAs derive from inverted repeat sequences and silence the relatively recessive *SP11* allele by inducing de novo DNA methylation of its promoter region [12,13]. *Smi* determines the dominance–recessivity relationships between class-I and class-II *SP11* alleles, and *Smi2* determines the linear relationships among the four class-II *SP11* alleles in a fashion dependent on the nucleotide sequences of the alleles. In particular, *Smi2* and its targets contain polymorphisms to control these complex linear relationships. Thus, we previously proposed a model in which interactions between sRNAs and targets based on sequence similarity control the complicated linear dominance hierarchy of male SI determinants [12].

Compared with cultivated *B. rapa*, *A. halleri*, a wild Brassicaceae species closely related to *Arabidopsis thaliana,* exhibits a more divergent hierarchical pollen dominance: (*S_20,_ S_13_*) > *S_12_* > *S_4_* > *S_3_* > *S_1_* [14]. A model describing how this male dominance hierarchy arose from multiple networks of sRNA target gains and losses has been proposed [9]. In addition, whether dominant *S* alleles carry a larger set of sRNAs, or whether a larger set of sRNA targets are carried by recessive *S* alleles, has been investigated [9].

*A. lyrata* also exhibits a complex pollen side dominance hierarchy among *S* haplotypes: class A2 (*S_39_*, *S_20_*, *S_50_*) > class A3 (*S_13_*, *S_16_*) > class B (*S_18_*, *S_14_*) > class A1 (*S_1_*) [14,15,16,17,18]. However, the mechanism underlying this dominance hierarchy, including whether it involves sRNA(s), was unknown. Here, we identified sRNAs controlling the linear dominance hierarchy in *A. lyrata*, and we propose a homology-dependent model to explain the dominance–recessivity interaction of *SP11* in *A. lyrata*.

## 2. Results

### 2.1. Analysis of the Dominance–Recessivity Hierarchy of the SP11 Alleles

**Figure 1:**
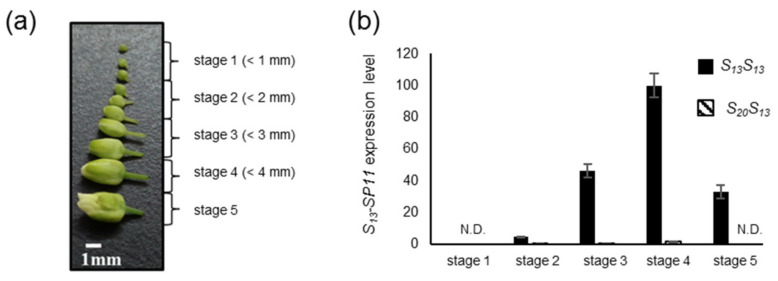
*S_13_-SP11* expression in anthers during each stage of development and dominance–recessivity relationship between the *S_20_* and *S_13_* haplotypes. (**a**) Developmental stages of *Arabidopsis lyrata* anthers: stage 1, <1 mm; stage 2, <2 mm; stage 3, <3 mm; stage 4, <4 mm; stage 5, the day before flowering. (**b**) RT-qPCR analysis of *S_13_-SP11* expression in *S_13_S_13_* homozygotes and *S_13_S_20_* heterozygotes. *Elf1α* was used as an endogenous reference gene. The results shown are means ± s.d. of 3 replicates. N.D., not detected.

*A. lyrata* exhibits a male side dominance hierarchy of *S* haplotypes, comprising class A2 (*S_39_*, *S_20_*, *S_50_*) > class A3 (*S_13_*, *S_16_*) > class B (*S_18_*, *S_14_*) > class A1 (*S_1_*) [14,15,16,17,18]. Here, for simplicity, we renamed these classes as follows: class IV (*S_20_*, *S_39_*, *S_50_*) > class III (*S_13_*, *S_16_*) > class II (*S_18_*, *S_14_*) > class I (*S_1_*). We focused on the dominance–recessivity relationship between *S_20_-SP11* (class IV) and *S_13_-SP11* (class III) (*S_20_* > *S_13_*) because the only previously reported data about such relationships in *A. lyrata* pollen was that the expression of *S_13_-SP11* is reduced in a *S_20_S_13_* heterozygote, as revealed by gel blot analysis and in situ hybridization [19]. First, we analyzed *SP11* expression in anthers from both *S_13_S_13_* homozygous and *S_20_S_13_* heterozygous plants at different stages of development. Developing buds were divided into five stages based on size (Figure 1a). Reverse transcription quantitative real-time PCR (RT-qPCR) analysis showed that *S_13_-SP11* transcripts started to accumulate at stage 2 (bud size: 1–2 mm) and showed maximum expression at stage 4 (bud size: 3–4 mm) in *S_13_S_13_* homozygotes (Figure 1b). Similarly, in a previous study, RNA gel blot analysis revealed that *SP11* expression in *B. rapa* anthers reaches a maximum before flower opening, when tapetum cells are intact [3]. In contrast, we found that *S_13_-SP11* transcript accumulation in *S_20_S_13_* heterozygotes was strongly suppressed (to approximately 1%) compared with that in *S_13_S_13_* homozygotes (Figure 1b), suggesting that *S_20_-SP11* is dominant over *S_13_-SP11.* This result is consistent with previously reported pollen phenotype data [14] and gel blot analysis of *S_13_-SP11* in *S_20_S_13_* heterozygotes [8]. Therefore, we focused on the molecular mechanism of the dominance–recessivity relationship among these *S* haplotypes.

### 2.2. Identification of sRNA and Its Precursor Genes in Class-IV S Haplotypes

**Figure 2:**
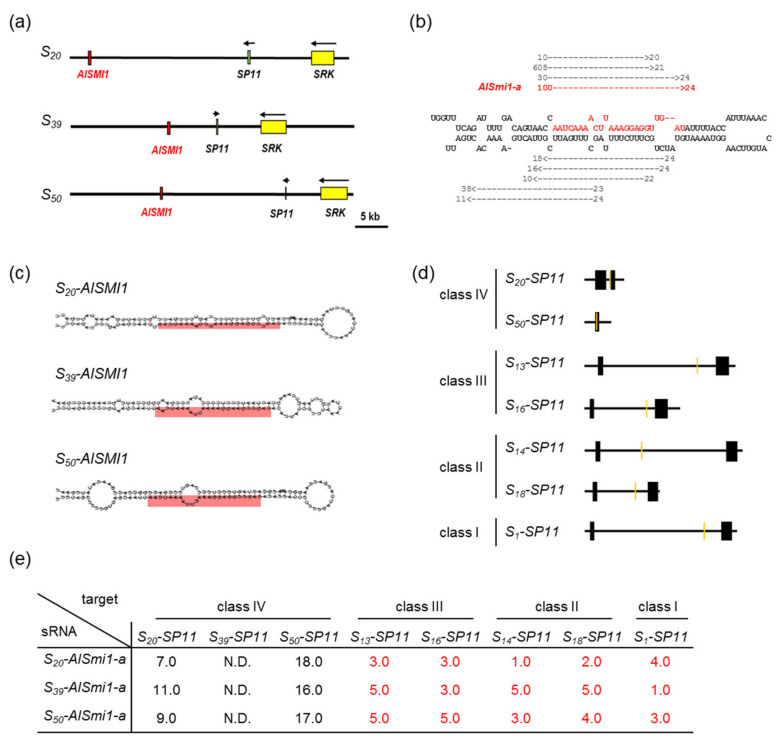
Identification of candidate sRNAs controlling the dominance–recessivity relationships: class IV > (class III, class II, class I). (**a**) Schematic diagrams of the *S* locus in the *S_20_* haplotype (HQ379628), *S_39_* haplotype (KJ772418–KJ772419), and *S_50_* haplotype (HQ379631). Green, yellow, and red boxes indicate *SP11*, *SRK*, and *AlSMI1*, respectively. (**b**) Massively parallel sequencing analysis of sRNA expression from the *AlSMI1* inverted repeat sequence. sRNAs that obtained at least 10 reads were mapped against the *AlSMI1* inverted repeat sequence. Arrows indicate mapped sRNAs and their directions. Numbers at the tips of arrows indicate the number of obtained sRNA reads, and numbers at the ends of arrows indicate the length of each sRNA (in nucleotides). Bases in red represent the 24-nt *AlSmi1-a* region. (**c**) Stem-loop structures of *AlSMI1* of the *S_20_*, *S_39_*, and *S_50_* haplotypes predicted by RNAfold [20]. The predicted mature 24-nt *AlSmi1-a* region is shown in red. (**d**) Schematic diagrams of the *S_20_*, *S_50_, S_13_, S_16_, S_14_, S_18_,* and *S_1_-SP11* genomic regions. Black boxes indicate exon regions of *SP11* genes, orange boxes indicate regions with high sequence similarity to *AlSmi1-a*. (**e**) Sequence similarity between *AlSmi1-a* and *SP11*. Each number shows the mispair score between the class IV (*S_20_*, *S_39_, S_50_*)**-***AlSmi1-a* and the *SP11* introns of class-IV, class-III, class-II, and class-I *S* haplotypes. N.D., not detected. Mispair scores < 5.5 are shown in red.

To identify sRNA candidates controlling the dominance–recessivity relationships between *S_20_-SP11* and *S_13_-SP11*, we performed in silico analysis of the *S*-locus genomic sequences of the *S_20_* haplotype. The aim was to identify sRNA candidate genes sharing high similarity with *S_13_-SP11* between the region 500 base pairs (bp) upstream from its translational initiation site and 500 bp downstream from its termination codon. We identified an inverted repeat sequence that we named *Arabidopsis lyrata SMI1* (*AlSMI1*) (Figure 2a), which shares high similarity with the *S_13_-SP11* intron region. *AlSMI1* is located 27.5 kbp downstream from *S_20_-SP11* and approximately 37 kbp from *S_20_-SRK*. We detected inverted repeat sequences similar to that of *S_20_-AlSMI1* in both the *S_39_* and *S_50_* haplotypes (class IV) (Figure 2a). 

We performed massively parallel sequencing to determine whether a 24-nucleotide (nt) sRNA is processed from *S_20_-AlSMI1* prior to *S_13_-SP11* expression. Since *S_13_-SP11* expression begins at stage 2 (Figure 1b), we analyzed sRNA from stage 1 and 2 anthers of *S_20_S_13_* heterozygotes and obtained 44,553,252 sRNA sequence reads. We identified 100 reads of the 24-nt sRNA *AlSmi1-a* (Figure 2b, Supplementary Table S1), which were processed from the *S_20_-AlSMI1* precursor. RNAfold [20] predicted that *S_20_-AlSMI1*, *S_39_-AlSMI1*, and *S_50_-AlSMI1* have hairpin-structured precursors (Figure 2c). We detected *AlSmi1-a* in the stem regions of *S_20_-AlSMI1*, *S_50_-AlSMI1*, and *S_39_-AlSMI1* (Figure 2c; Supplementary Figure S1).

We also analyzed other *S*-locus genomic sequences of the *S_16_* (class III), *S_14_* and *S_18_* (class II), and *S_1_* (class I) haplotypes, which are recessive to the *S_20_* haplotype in the pollen dominance hierarchy. We searched the sequences of the *S_16_*-, *S_18_*-, *S_14_*-, and *S_1_-SP11* genes ±500 bp and determined that only the *AlSMI1-a* region in the *S* locus of the *S_20_* haplotype shares high similarity with these *SP11* genes (Figure 2d; Supplementary Figures S1 and S2).

To evaluate the activity of *AlSmi1-a*, we calculated mispair scores [21] between the sRNA and *S_13_-SP11*. Although the mispair score system was originally developed to predict whether a 21-nt microRNA (miRNA) cuts its target, it also efficiently predicted the 24-nt *Smi* and *Smi2* targets [12]. Mispair scores represent the number of mismatches of the complementary sites between an sRNA and its target, thus, high complementarity between an sRNA and its target leads to a lower mispair score. In our previous study, the SI phenotypes were not observed in transgenic *B. rapa* plants expressing *Smi/Smi2* with high sequence similarity with their own *SP11* (mispair scores ≤ 5.5), exhibiting DNA methylation at *SP11* and its silencing [11,12]. On the other hand, transgenic *B. rapa* plants expressing *Smi2* showing low similarity with their own *SP11* (mispair scores ≥ 6.5), exhibited unchanged SI phenotypes [12]. Therefore, the mispair scores between dominant *Smi* or *Smi2* and the target sites of recessive *SP11* were ≤5.5. Here, *S_20_*-*AlSmi1-a* had a mispair score of <3.0 against the class-III *SP11* intron, <2.0 against the class-II *SP11* intron, and 4.0 against the class-I *SP11* intron (Figure 2e; Supplementary Figure S2). Both *S_39_-AlSmi1-a* and *S_50_-AlSmi1-a* had mispair scores of <5.0 against the *SP11* intron regions of all recessive *S* haplotypes (Figure 2e; Supplementary Figure S2), suggesting that *AlSmi1-a* potentially targets *SP11* in all recessive *S* haplotypes with the same dominance relationship: class IV > (class III, class II, class I). On the other hand, although class-IV *S* haplotypes (*S_20,_ S_50_*) had target sites for *AlSmi1-a* (Figure 2d, Supplementary Figure S2), all *AlSmi1-a* sequences had mispair scores > 7.0 against these target sites (Figure 2e; Supplementary Figure S2).

Based on these results, we suggest that *AlSmi1-a* is the only sRNA controlling the class IV > (class III, class II, class I) dominance hierarchy. On the other hand, *AlSMI1* genes are also conserved in class-III and class-II *S* locus with polymorphisms (Supplementary Figure S1).

### 2.3. Identification of sRNA and Its Precursor Genes in Class-II S Haplotypes

**Figure 3:**
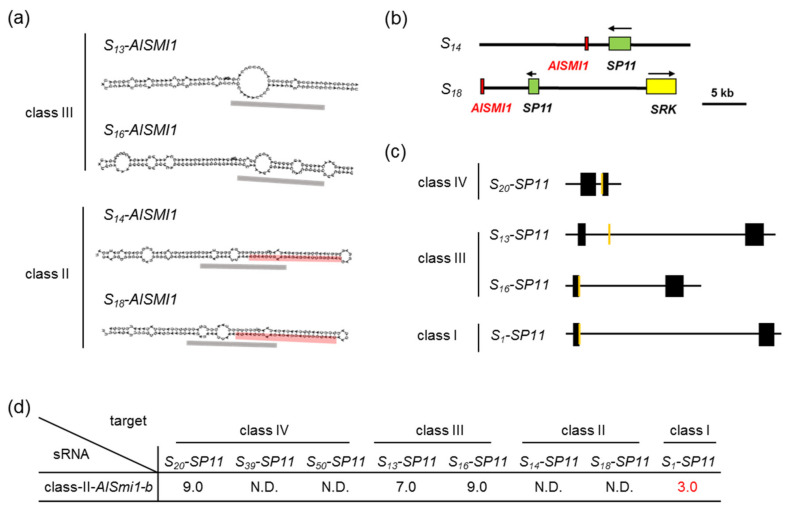
Identification of sRNAs controlling the dominance–recessivity relationships: class II > class I. (**a**) Stem-loop structures of the *S_13_, S_16_, S_14_,* and *S_18_* haplotypes of *AlSMI1* predicted by RNAfold [20]. Black dashed boxes indicate 24-nt snRNAs corresponding to class-III and class-II *AlSmi1-a.* The predicted 24-nt mature *AlSmi1-b* is shown in red. (**b**) Schematic diagrams of the *S* locus in the *S_14_* haplotype (KJ772406) and *S_18_* haplotype (KJ772412). Green, yellow, and red boxes indicate *SP11, SRK*, and *AlSMI1*, respectively. (**c**) Schematic diagrams of the *SP11* genomic regions of the *S_20_*, *S_13_*, *S_16_*, and *S_1_* haplotypes. Black boxes indicate exon regions of *SP11,* orange boxes indicate the target regions homologous to *AlSmi1-b*. (**d**) Sequence similarity between *AlSmi1-b* and *SP11*. Numbers are the mispair scores between class-II *AlSmi1-b* and class-IV, class-III, class-II, and class-I *S* haplotypes of *SP11*. N.D., not detected. Mispair scores < 5.5 are shown in red.

Next, we focused on the sequence polymorphisms of *AlSMI1* among class-III and class-II *S* haplotypes. *AlSMI1* genes from the *S_13_* and *S_16_* haplotypes (class III) and the *S_18_* and *S_14_* haplotypes (class II) have similar inverted repeat structures. However, the 24-nt *S_20_-AlSmi1-a* sequence is not conserved among either class-III or class-II *S* haplotypes (Figure 3a; Supplementary Figure S1). To analyze whether *AlSmi1* is processed in class-III *S* haplotypes, we mapped reads obtained by the massively parallel sequencing of *S_20_S_13_* against the *S_13_-AlSMI1* inverted repeat sequence. No reads were mapped onto the *S_13_-AlSMI1* precursor. These results suggest that *S_13_-AlSMI1* is not processed into 24-nt sRNAs and that *AlSMI1* is not functional in this class-III *S* haplotype (Figure 3a).

Interestingly, *S_18_* and *S_14_-AlSMI1* (class II) contain another 24-nt sequence (*AlSmi1-b*) that shares high similarity with a relatively recessive *S_1_-SP11* (class I) exon junction (mispair score 3.0) (Figure 3a–d; Supplementary Figures S1 and S2). This suggests that sRNA processed from this region might be involved in the dominance relationship between class II and class I (class II > class I). *AlSmi1-b* had a mispair score > 7.0 against *S_20_-SP11* (class IV) and *S_13,_ S_16_-SP11* (class III), consistent with dominance hierarchy (class IV and class III > class II) (Figure 3c,d; Supplementary Figure S2). These results suggest that the interaction between *AlSmi1-a* from class-IV *S* haplotypes and its target site at the *SP11* intron of recessive class-III, class-II, and class-I *S* haplotypes controls the dominance relationships among these *S* haplotypes. On the other hand, the interaction between *AlSmi1-b* from class-II *S* haplotypes and its target site at the *SP11* exon junction of recessive class-I *S* haplotypes controls the dominance relationships among these *S* haplotypes.

### 2.4. Identification of sRNA and Its Precursor Genes in Class-III S Haplotypes

**Figure 4:**
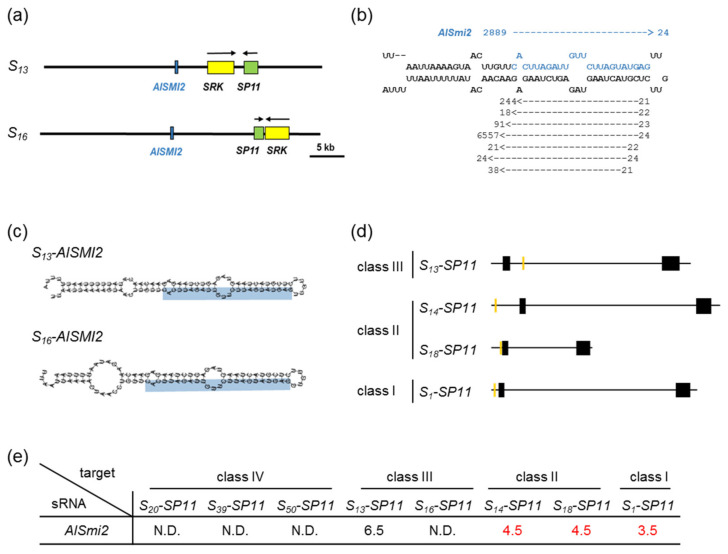
Identification of the candidate sRNAs controlling dominance–recessivity relationships: class III > (class II, class I). (**a**) Schematic diagrams of the *S* locus in the *S_13_* haplotype (ADBK01001387) and the *S_16_* haplotype (HQ379629). Green, yellow, and blue boxes indicate *SP11, SRK*, and *AlSMI2,* respectively. (**b**) Massively parallel sequencing analysis of sRNA expression from the *S_13_-AlSMI2* inverted repeat sequence. sRNAs obtained from more than 10 reads were mapped against the *S_13_-AlSMI2* inverted repeat sequence. Arrows indicate mapped sRNAs and their directions. Numbers at the tips of arrows indicate the obtained read numbers, and numbers at the ends of arrows indicate the base length of each sRNA. Bases in blue indicate the 24-nt *AlSmi2* region. (**c**) Stem-loop structures of *AlSMI2* of the *S_13_* and *S_16_* haplotypes predicted by RNAfold [20]. The predicted 24-nt mature *AlSmi2* is shown in blue. (**d**) Schematic diagrams of the *SP11* genomic regions of the *S_13_, S_14_, S_18_,* and *S_1_* haplotypes. Black boxes indicate exon regions of *SP11*, orange boxes indicate the target region homologous to *AlSmi2*. (**e**) Sequence similarity between *AlSmi2* and *SP11*. Each number indicates the mispair score between class-III *AlSmi2* and class-IV, class-III, class-II, and class-I *S* haplotypes of *SP11*. N.D., not detected. Mispair scores < 5.5 are shown in red.

Since processed *S_13_-AlSmi1* was not detected in *S_20_S_13_* heterozygotes, we searched for other sRNAs that could control the dominance relationship class III > (class II and class I). We searched the *S* locus genomic sequence of the *S_13_* haplotype, which shares high similarity with class-II *S_14_*- and *S_18_-SP11* and class-I *S_1_-SP11* genes ± 500 bp. We identified another inverted repeat sequence with high similarity to the 30-bp region upstream from the translation initiation sites of class-I and class-II *SP11* genes, which we named *A. lyrata SMI2* (*AlSMI2*) (Figure 4a). A database search identified the *AlSMI2* sequence only in the class-III *S_13_* and *S_16_* haplotypes (Figure 4a).

We mapped sRNA sequence reads derived from the anthers of *S_20_S_13_* heterozygotes against the *S_13_-AlSMI2* region and identified 2,889 *AlSmi2* reads sharing high similarity with the 5’ upstream region of *SP11* (Figure 4b; Supplementary Table S1). RNAfold [20] predicted that *S_13_-AlSMI2* and *S_16_-AlSMI2* had hairpin-structured precursors (Figure 4c). The mature 24-nt *AlSmi2* sequence was identified in the stem regions of *S_13_-AlSMI2* and *S_16_-AlSMI2* (Figure 4c).

*AlSmi2* shares high similarity with the 5’ regions of the relatively recessive haplotypes *S_14_*- and *S_18_-SP11* (class II) and *S_1_-SP11* (class I), with mispair scores < 4.5 (Figure 4d,e; Supplementary Figure S2). Although *S_13_-SP11* intron contains a candidate target site of *AlSmi2*, it showed low similarity with this sequence (mispair score 6.5) (Figure 4d,e; Supplementary Figure S2). These results suggest that *AlSmi2* controls the dominance relationship class III > (class II, class I) as a result of its high similarity (mispair score < 4.5) with the *SP11* sequences of relatively recessive *S* haplotypes.

### 2.5. Quantification of a 24-nt sRNA and Its Precursor

**Figure 5:**
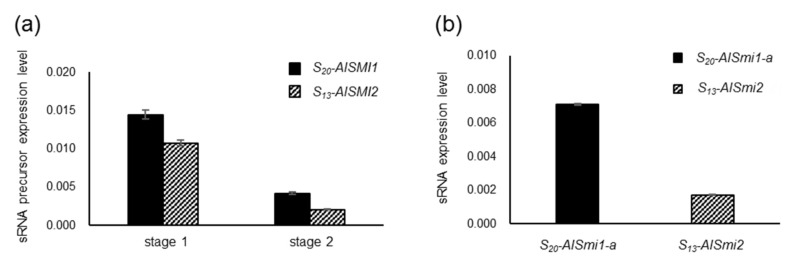
Accumulation of sRNA precursors and a 24-nt sRNA in early stage anthers of *S_20_S_13_* heterozygotes. (**a**) RT-qPCR of *S_13_-AlSMI2* and *S_20_-AlSMI1* precursors at stages 1 and 2 in *S_20_S_13_* heterozygotes. *GAPDH* was used as an endogenous reference gene. The results shown are means ± s.d. of 3 replicates. (**b**) Quantification of *S_13_-AlSmi2* and *S_20_-AlSmi1* in mixtures of stage 1 and 2 anthers in *S_20_S_13_* heterozygotes via stem-loop RT-PCR analysis. *miR166* was used as an endogenous reference gene. The results shown are means ± s.d. of 3 replicates.

To confirm the expression of *S_20_-AlSMI1* and *S_13_-AlSMI2*, which are precursors of *S_20_-AlSmi1-a* and *S_13_-AlSmi2,* respectively, in *S_20_S_13_* heterozygotes, we performed RT-qPCR (Figure 5a). In *B. rapa*, the expression of *Smi1* and *Smi2* is induced before the initiation of *SP11* transcription [13]. Since *SP11* transcription is initiated at stage 2 in *A. lyrata* (Figure 1b), we analyzed *S_20_-AlSMI1* and *S_13_-AlSMI2* precursors in stage 1 and 2 anthers. We detected strong accumulation of both the *S_20_-AlSMI1* and *S_13_-AlSMI2* precursors at stage 1, which then declined by stage 2 (Figure 5a). This expression pattern suggests that *S_20_-AlSMI1* and *S_13_-AlSMI2* might act to suppress *SP11*. Additionally, we analyzed the expression of mature 24-nt *S_20_-AlSmi1-a* and *S_13_-AlSmi2* in early stage anthers (stages 1–2) of *S_20_S_13_* heterozygotes via stem-loop RT-qPCR of RNA samples [22] (Figure 5b). The results suggest that the precursors of these sRNAs and mature 24-nt sRNAs are expressed in vivo.

## 3. Discussion

**Figure 6:**
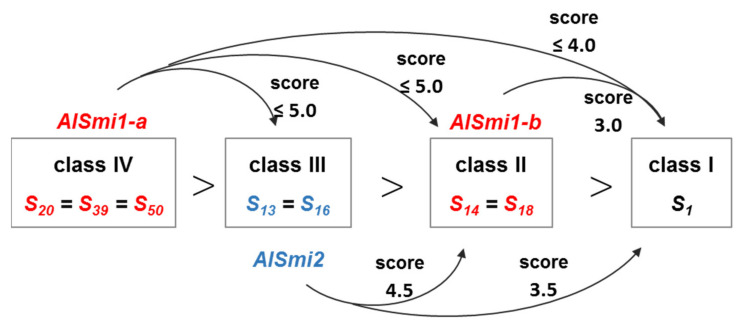
Regulatory network of the identified sRNAs and their predicted targets in *A. lyrata.* Class-IV *S_20_-SP11* is dominant over class-III *S_13_-SP11* (Figure 1). *AlSmi1-a* from class-IV *S* haplotypes is homologous to *SP11* from recessive *S* haplotypes (classes III, II, and I), showing a mispair score < 5.0 (Figure 2). *AlSmi1-b* predicted from class-II *S* haplotypes is homologous to a class-I *SP11*, showing a mispair score < 3.0 (Figure 3). *AlSmi2* from class-III *S* haplotypes is homologous to *SP11* from relatively recessive *S* haplotypes (classes II and I), showing a mispair score < 4.5 (Figure 4). Both precursors and mature 24-nt sRNA of *AlSmi1-a* and *AlSmi2* were detected in vivo (Figure 5).

In this study, we identified two sRNAs homologous to *SP11* genes in *A. lyrata*. The accumulation of the *S_13_-SP11* transcript in *S_20_S_13_* heterozygotes was strongly suppressed (to approximately 1%) compared with that in *S_13_S_13_* homozygotes (Figure 1b), suggesting that class-IV *S_20_-SP11* is dominant over class-III *S_13_-SP11. AlSmi1-a* from class IV is thought to be the only sRNA that controls the relationship class IV > (class III, class II, class I) (Figure 2a–e). Class-II and class-III *AlSMI1* gene sequences with polymorphisms are also conserved (Supplementary Figure S1). Class II-specific *AlSmi1-b* shares high similarity with a relatively recessive *S_1_-SP11* (class I) exon junction (mispair score 3.0) (Figure 3a–d; Supplementary Figure S1 and S2), suggesting that sRNA processed from this region might be involved in the dominance relationship between class-II and class-I sRNAs (class II > class I). Another sRNA, *AlSmi2*, which shares high similarity with *SP11* genes of relatively recessive *S* haplotypes, is involved in the dominance relationship class III > (class II, class I) (Figure 4a–e). The accumulation of both *S_20_-AlSMI1* and *S_13_-AlSMI2* precursors was detected (Figure 5a), and mature 24-nt sRNAs of *S_20_-AlSmi1-a* and *S_13_-AlSmi2* were expressed in vivo (Figure 5b). These results suggest that the linear dominance hierarchy in *A. lyrata* is controlled by two polymorphic sRNAs, *AlSmi1* and *AlSmi2* (Figure 6). 

*AlSmi1* and *AlSmi2* correspond to *mirS3* and *mir1887* in *A. halleri*, respectively [9]. A species-wide survey of sequence diversity revealed that a large fraction of alleles at the pistil side SI determinant *SRK* are trans-specifically shared between *A. lyrata* and *A. halleri* [23]. Our findings suggest that not only *SRK* but also other *S-*locus genes, including sRNAs, are shared between *A. lyrata* and *A. halleri.* These sRNAs are conserved in both species in the regulation of pollen side dominance. A pollen side linear dominance hierarchy controlled by sRNAs has also been identified in *B. rapa* [11,12], pointing to a common sRNA-based dominance–recessivity mechanism in Brassicaceae plants.

Furthermore, our data suggest that the dominance–recessivity mechanism in both *B. rapa* and *A. lyrata* is explained by the sequence homology between sRNA and its target. In our previous report, the sRNA from the dominant allele had a mispair score < 5.5 against the target region of recessive *SP11*. In the current study, *AlSmi1* and *AlSmi2* in *A. lyrata* also had mispair scores < 5.0 against relatively recessive *SP11s* (Figure 2e, Figure 3d, Figure 4e and Figure 6; Supplementary Figure S2). Concurrently, these sRNAs had a mispair score > 6.5 against self- and relatively dominant *SP11s* (Figure 2e, Figure 3d and Figure 4e; Supplementary Figure S2). Two models have been examined in *A. halleri*: (1) dominant *S* alleles carry a larger set of sRNAs; and (2) recessive *S* alleles carry a larger set of sRNA targets [9]. Here, we identified only one sRNA, *AlSmi1-a,* from the most dominant *S* haplotype (class IV), and found that recessive *S* haplotypes did not contain a larger number of sRNA targets (Figure 2b, Figure 3c and Figure 4d). These results strongly suggest that the polymorphic dominance modifier model we proposed for *B. rapa* also explains the dominance relationships in *A. lyrata*. Further analysis of the role of the interactions between sRNAs and their target regions should provide support for this model.

Similar complex dominance–recessivity relationships can be observed in other SI plants of the Asteraceae and Convolvulaceae [24,25]. Moreover, dominance relationships have been reported among mimicry genes in butterflies [26] and among genes in various plants and animals [27,28,29,30]. Further studies are needed to determine whether our model can explain these complicated dominance–recessivity networks and monoallelic gene expression.

## 4. Materials and Methods

### 4.1. Plant Materials

*A. lyrata SaSb* seeds were a gift from Y. Takada (Tohoku University). For all experiments, floral buds from clonally propagated progenies of a single individual were used. The plants were grown in a growth chamber under long-day conditions (16 h light/8 h dark).

### 4.2. RT-qPCR of SP11

Total RNA was isolated from the anthers of *S_13_S_13_* homozygotes and *S_20_S_13_* heterozygotes at each stage of development and used for RT-qPCR, as previously described [13]. The *S_13_-SP11* region was amplified using specific primers: forward primer, 5’-AGCCATGTTCAAGGAATGGAAGA-3’; reverse primer, 5’-TTGTTGCCATCCTCCGTAAGGTC-3’. *Elf1α* was used as an endogenous reference gene and amplified using specific primers: forward primer, 5’-TGGTGACGCTGGTATGGTTA-3’; reverse primer, 5’-GGTCTGCCTCATGTCCCTAA-3’.

### 4.3. Prediction of Inverted Repeat Sequence Regions 

Inverted repeat sequences were searched from the *S*-locus sequence data of each haplotype: *S_20_* (HQ379628)*, S_39_* (KJ772415–KJ772419), *S_50_* (HQ379631)*, S_13_* (ADBK01001387)*, S_16_* (HQ379629)*, S_14_* (KJ772405–KJ772407), *S_18 _*(KJ772408–KJ772414), and *S_1_* (KJ772401–KJ772404). The *S* locus was defined as the region between *PUB8* (*At4g21350*) and *ARK3* (*At4g21380*), as described previously [15]. First, each *SP11* gene containing both upstream and downstream 500-bp fragments was divided into 500-bp fragments overlapping every 30-bp region. These fragments were used as queries in BLAST searches against the *S* loci of the *S_20_* and *S_13_* haplotypes. Sequences showing *E-*values < 1 × 10^−3^ were further analyzed. The secondary structures of the precursors from the obtained inverted repeat were predicted using RNAfold [20]. Hairpin-structured miRNA-like sequences having low energy values under −30 kcal mol^−1^ were screened.

### 4.4. Small RNA Sequencing and Mapping 

Total RNA containing the sRNA fraction was isolated from a mixture of stage 1 and 2 anthers using a *mir*Vana miRNA Isolation Kit (Ambion/Thermo Fisher Scientific, Waltham, MA, USA). Preparation of the small RNA library, Illumina GAII sequencing, and adaptor trimming of sequencing reads were performed at Hokkaido System Science Co., Ltd (Sapporo, Japan). sRNA sequences with more than 10 reads were mapped against the *S* locus of the *S_20_* haplotype (HQ379628) and the *S_13_* haplotype (ADBK01001387). We also mapped the obtained reads against the predicted precursors of *AlSMI1* and *AlSMI2* using the *Bowtie* program [31]. The mispair score was calculated as described [21]: mismatches were scored as 1, and G:U pairs were scored as 0.5. Mismatched and G:U pair scores within the core segment were doubled.

### 4.5. RT-qPCR of sRNA Precursor 

Total RNA was isolated from anthers of *S_20_S_13_* heterozygotes at each stage of development using an RNeasy Mini Kit (QIAGEN GmbH, Hilden, Germany) and used for RT-qPCR, as previously described [13]. Each sRNA precursor region was amplified using specific primers: *S_20_-AlSMI1* forward primer, 5’-AGCAATGGTTTCAGATTTTGACAGTAACC-3’; *S_20_-AlSMI1* reverse primer, 5’-AGATACATTTTACCTTGAACATGGTTTAAATGG-3’; *S_13_-AlSMI2* forward primer, 5’-TTAATTAAAAGTAACTTGTTCACTTAGATTGTTCTTAG-3’; *S_13_-AlSMI2* reverse primer, 5’-ATGTTGTTCTCTTAGACTCTACTTAGTACG-3’. *GAPDH* was used as an endogenous reference gene and amplified using specific primers: forward primer, 5’-GACCTTACTGTCAGACTCGAG-3’; reverse primer, 5’-CGGTGTATCCAAGGATTCCCT-3’.

### 4.6. Stem-Loop RT-PCR 

Quantification of 24-nt *S_20_-AlSmi1* and *S_13_-AlSmi2* sRNAs was performed as previously described [22]. sRNAs were isolated from anthers (mixture of stages 1 and 2) from *S_20_S_13_* heterozygotes using a *mir*Vana miRNA Isolation Kit (Ambion/Thermo Fisher Scientific, Waltham, MA, USA) and reverse transcribed using a TaqMan MicroRNA Reverse Transcription Kit (Applied Biosystems/Thermo Fisher Scientific, Waltham, MA, USA) with 24-nt sRNA-specific RT primers: *S_20_-AlSmi1* RT-forward primer, 5’-GTTGGCTCTGGTGCAGGGTCCGAGGTATTCGCACCAGA-3’; *S_13_-AlSmi2* RT-forward primer, 5’-GTTGGCTCTGGTGCAGGGTCCGAGGTATTCGCACCAGA-3’. The reverse transcription products were quantified using Light Cycler 480 Probes Master Mix (Roche, Basel, Switzerland) and Universal Probe Library #21 (Roche) with small RNA-specific primers and universal primers: *S_20_-AlSmi1* forward primer, 5’-CGGCGGCAATCAAAACTTAAAGGAG-3’; *S_13_-AlSmi2* forward primer, 5’-CGGCGGCCACTTAGATTGTTCTTAG-3’; universal primer, 5’-GTGCAGGGTCCGAGGT-3’. *miR166* was used as an endogenous reference miRNA and was amplified using specific primers: RT-forward primer, 5’-GTTGGCTCTGGTGCAGGGTCCGAGGTATTCGCACCAGAGCCAACGGGGAA-3’; forward primer, 5’-CAGCATCGGACCAGGCTTCA-3’.

## Data Availability

Sequence data for *S_20_*-*AlSMI1* and *S_13_*-*AlSMI2* have been deposited in DDBJ under accession numbers LC629453 and LC629454.

## Supplementary Materials

Supplementary materials can be found at https://www.mdpi.com/article/10.3390/ijms22136990/s1.
