# Supplementary material for: Homology-Based Interactions between Small RNAs and Their Targets Control Dominance Hierarchy of Male Determinant Alleles of Self-Incompatibility in Arabidopsis lyrata
Source: Int J Mol Sci. 2021 Jun 29;22(13):6990. doi: 10.3390/ijms22136990 (PMC8268441; doi:10.3390/ijms22136990)
Supplement: Supplementary file 1 [file ijms-22-06990-s001.zip › ijms-1224018-supplementary.pdf]

Supplementary Materials

Homology-Based Interactions between Small RNAs and Their Targets Control Dominance Hierarchy of Male Determinant Alleles of Self-Incompatibility in *Arabidopsis lyrata*

Shinsuke Yasuda [1,†,‡], Risa Kobayashi [1,‡], Toshiro Ito [1], Yuko Wada [1,*] and Seiji Takayama [2,*]

1 Division of Biological Sciences, Nara Institute of Science and Technology, Nara 630-0192, Japan; shinsuke-yasuda@takii.co.jp (S.Y.); kobayashi.risa.ki7@bs.naist.jp (R.K.); itot@bs.naist.jp (T.I.)

2 Department of Applied Biological Chemistry, Graduate School of Agricultural and Life Sciences, The University of Tokyo, Tokyo 113-8657, Japan

* Correspondence: yu-wada@gtc.naist.jp (Y.W.); a-taka@g.ecc.u-tokyo.ac.jp (S.T.)

† Present address: Plant Breeding and Experiment Station, Takii and Company, Limited, Konan, Shiga 520-3231, Japan

‡ These authors contributed equally to this work.

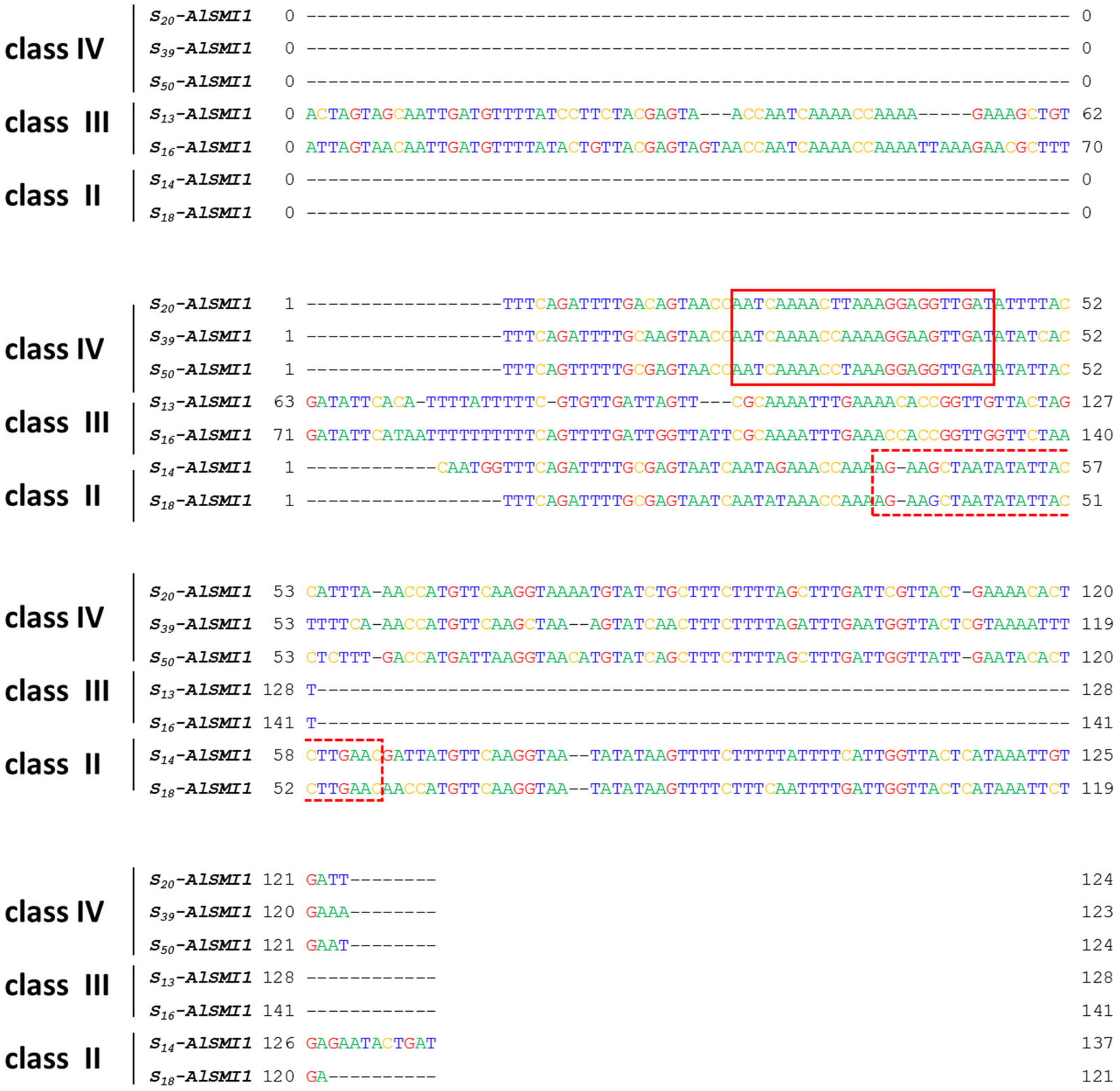

Supplementary Figure S1. Sequence alignment of the inverted repeat sequences of *AlSmi1*. Alignment of *S*20, *S*50, *S*39, *S*13, *S*16, *S*14, and *S*18 haplotypes of *AlSmi1*. Class-IV *AlSmi1-a* sequences and predicted *AlSmi1-b* sequences are highlighted by solid and dashed red boxes, respectively.

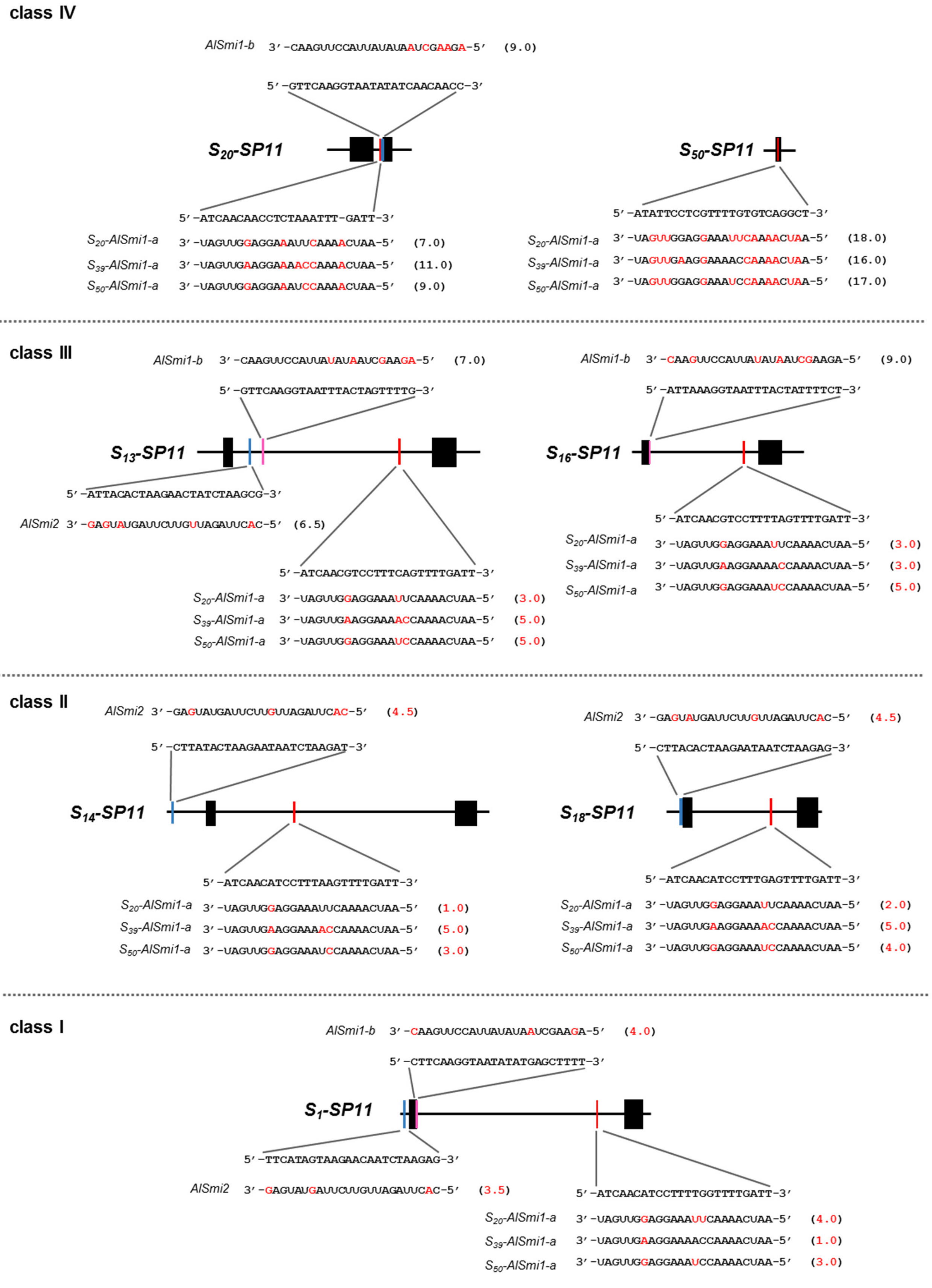

Supplementary Figure S2. Genomic regions of the class-IV, class-III, class-II, and class-I *S* haplotypes of *SP11*, and sequence similarity between sRNAs and their *SP11* target sites. Schematic diagram of haplotypes S_{20}, S_{50}, S_{13}, S_{16}, S_{14}, S_{18}, and S_{1}. Sequences of S_{20}-*AlSmi1-a*, S_{39}-*AlSmi1-a*, S_{50}-*AlSmi1-a*, *AlSmia-b*, and *AlSmi2* and their sRNA target sites in *SP11* are shown. Black boxes indicate *SP11* exon regions. Red, magenta, and blue boxes indicate target sites of *AlSmi1-a*, *AlSmi-b*, and *AlSmi2*, respectively. Bases in red indicate mismatches with each *SP11* target site. Numbers in parentheses indicate the mispair scores between each sRNA and the *SP11* target site. Mispair scores <5.5 are shown in red.

Supplementary Table S1. Number of sequence reads obtained.

sRNA	Obtained Reads Number
S_{20}-AlSmi1-a	100
S_{13}-AlSmi2	2889

Results of massively parallel sequencing of *$S_{20}S_{13}$* heterozygotes. A total of 44,553,252 bp sequence reads were obtained.
